# P027 Balancing bleeding and thrombosis: delayed recognition of triple-positive antiphospholipid syndrome in refractory ITP

**DOI:** 10.1093/rap/rkag113.057

**Published:** 2026-11-03

**Authors:** Kelsey Campbell, Jordan Burgess

**Affiliations:** NHS GGC, Glasgow, United Kingdom; NHS GGC, Glasgow, United Kingdom

## Abstract

**Introduction:**

This case describes the delayed diagnosis of triple antibody-positive antiphospholipid syndrome (APS) in a patient with longstanding refractory immune thrombocytopenia (ITP), illustrating the diagnostic and therapeutic challenges posed by competing risks of thrombosis and bleeding. Chronic severe thrombocytopenia complicated both recognition of APS and subsequent anticoagulation decisions, resulting in multiple recurrent thrombotic events before the diagnosis was confirmed. The case highlights a missed opportunity for earlier investigation following an initial thrombotic event and explores the complexities of balancing treatment for refractory ITP with appropriate long-term anticoagulation in a patient at exceptionally high thrombotic risk.

**Case description:**

Patient X is a 59-year-old gentleman who has been under haematology follow-up for approximately 16 years. Originally from Poland, he was diagnosed with immune thrombocytopenia (ITP) prior to relocating to the UK. His disease has been highly refractory despite multiple treatment modalities, including mycophenolate mofetil, rituximab, eltrombopag, romiplostim, azathioprine and splenectomy. His medical history also includes poorly controlled type 2 diabetes mellitus, hypertension, obesity and previous alcohol excess.

Throughout his care in Glasgow, platelet counts remained persistently between 0–20 × 10⁹/L for much of the disease course despite treatment with corticosteroids, intravenous immunoglobulin (IVIg), vincristine, ciclosporin, romiplostim, eltrombopag and avatrombopag. He experienced multiple hospital admissions with bleeding complications, including epistaxis, haemorrhoidal bleeding and oral blood blisters.

His first deep vein thrombosis (DVT) occurred in May 2015. Investigation demonstrated an isolated prolonged activated partial thromboplastin time (APTT), highly suggestive of antiphospholipid syndrome (APS). An antiphospholipid antibody screen was requested but the sample clotted and was never repeated, representing a missed diagnostic opportunity. Anticoagulation with apixaban proved difficult because of recurrent interruptions secondary to profound thrombocytopenia.

He subsequently developed superficial thrombophlebitis in 2017 and 2018, followed by an extensive superior vena cava and right internal jugular vein thrombosis in 2019. Repeat APS testing demonstrated lupus anticoagulant positivity; however, warfarin was deferred because of the perceived high bleeding risk associated with persistent severe thrombocytopenia.

In September 2023, he presented with a large saddle pulmonary embolism with right ventricular strain, followed shortly by a right subclavian artery embolism despite therapeutic-dose apixaban. He was transitioned to warfarin (initial target INR 2.5), with repeat testing confirming triple antibody-positive APS. Despite anticoagulation, he later developed a left popliteal vein thrombosis extending into the distal femoral vein, necessitating an increased target INR of 3.5.

**Discussion:**

This case demonstrates the diagnostic complexity of APS in a patient with longstanding refractory immune thrombocytopenia. Although recurrent thrombotic events should prompt consideration of APS, severe thrombocytopenia and the competing risk of bleeding complicated both investigation and management. The inability to confirm APS following the initial DVT in 2015, when an antiphospholipid antibody sample clotted and was not repeated, may have delayed diagnosis and influenced subsequent therapeutic decisions.

Management required continual assessment of the balance between thrombosis and haemorrhage. Corticosteroids remained a potential treatment for refractory ITP but were used cautiously because of obesity and poorly controlled diabetes mellitus. Similarly, thrombopoietin receptor agonists (eltrombopag, romiplostim and avatrombopag), while effective at increasing platelet counts and reducing bleeding risk, are recognised to increase thrombotic risk. ITP itself is also associated with paradoxical thrombosis through increased platelet turnover and procoagulant platelet microparticles. Additional contributing factors included obesity, previous immobility associated with illness and frequent travel.

Following lupus anticoagulant positivity in 2019, earlier transition to warfarin was considered, particularly given emerging evidence demonstrating inferior outcomes with direct oral anticoagulants in high-risk APS. However, persistent severe thrombocytopenia and recurrent bleeding episodes represented legitimate concerns regarding the safety of vitamin K antagonist therapy. Even after confirmation of triple antibody-positive APS and transition to warfarin, recurrent thrombosis necessitated escalation to a higher INR target, highlighting the aggressive thrombotic phenotype associated with high-risk APS.

This case illustrates the importance of maintaining a high index of suspicion for APS in patients with unexplained thrombosis, even in the presence of severe thrombocytopenia, and emphasises the challenges of balancing haemorrhagic and thrombotic risks.

**Key learning points:**

• APS should be considered in any patient presenting with unprovoked venous or arterial thrombosis, particularly when accompanied by an isolated prolonged activated partial thromboplastin time.

• A negative or incomplete antiphospholipid antibody investigation should not exclude the diagnosis; persistent positivity on two occasions at least 12 weeks apart is required for confirmation.

• ITP does not protect against thrombosis. Patients with refractory ITP may have multiple prothrombotic risk factors, including the disease itself and treatments such as thrombopoietin receptor agonists and intravenous immunoglobulin.

• Warfarin remains the preferred anticoagulant for thrombotic APS, particularly in patients with triple antibody positivity, while higher INR targets may be appropriate in selected patients with recurrent thrombosis despite therapeutic anticoagulation.

• Management of patients with concurrent APS and severe ITP requires individualised multidisciplinary decision-making to balance the competing risks of thrombosis and bleeding.

**Learning objectives:**

• As a Clinical Fellow in Haematology, I hope to broaden my understanding of autoimmune disease through greater exposure to rheumatology, particularly conditions that overlap with haematological practice such as antiphospholipid syndrome. Presenting this case would also provide an opportunity to develop my academic presentation skills, discuss optimal anticoagulation strategies in patients with concurrent severe thrombocytopenia and improve my professional portfolio. I anticipate that this case will hopefully stimulate discussion regarding multidisciplinary management and the challenges of diagnosing APS in atypical clinical settings.

